# On the integration of collective motion and temporal synchrony in animal collectives

**DOI:** 10.1186/s40462-025-00573-2

**Published:** 2025-07-01

**Authors:** Guy Amichay, Máté Nagy

**Affiliations:** 1 Centre for the Advanced Study of Collective Behaviour, University of Konstanz, Konstanz, Germany; 2 Department of Collective Behaviour, Max-Planck Institute of Animal Behavior, Konstanz, Germany; 3 Department of Biology, University of Konstanz, Konstanz, Germany; 4 Department of Engineering Sciences and Applied Mathematics, Northwestern University, Evanston, IL USA; 5 Northwestern Institute for Complex Systems, Northwestern University, Evanston, IL USA; 6 National Institute for Theory and Mathematics in Biology, Northwestern University, Chicago, IL USA; 7 MTA-ELTE Lendület Collective Behaviour Research Group, Hungarian Academy of Sciences, Budapest, Hungary; 8 Department of Biological Physics, Eötvös Loránd University, Budapest, Hungary

**Keywords:** Self-organization, Collective motion, Synchronization, Active matter, Coupled oscillators, Vicsek, Kuramoto

## Abstract

Animal groups come in diverse forms—from fish schools swimming in unison to crickets chirping in synchrony. Although these behaviors may seem considerably different to one another, they share a common mathematical core, and can therefore be considered in a unified manner. We discuss the commonalities and differences by synthesizing existing literature from both fields, encompassing both theoretical and empirical advances. We emphasize the crucial role of mixing, induced by individual movements, as a main differentiating factor. Along the way, we propose promising future directions for achieving a more comprehensive understanding of self-organized collective behavior.

## Introduction

Half a century ago, Yoshiki Kuramoto published what is now known as the Kuramoto model [1]. Two decades later, Tamás Vicsek and his colleagues introduced the so-called Vicsek model [2]. Both were pivotal in creating new research fields. Given their anniversaries, it is a fitting moment to revisit their legacy, and to reflect on opportunities for a deeper dialogue between the fields.

In the study of animal collective behavior, researchers often seek universal concepts that span scales and taxa [3]. A common goal is to understand how individuals coordinate via relatively local interactions to achieve large-scale collective order. In this context, we can view various systems, such as flying bird flocks or synchronous firefly swarms, as sharing common principles, despite their different manifestations. In bird flocks, organization occurs both spatially and temporally, as birds move in the same direction at the same time. We will refer to this as *collective motion* or *spatial alignment*. In contrast, firefly swarms exhibit temporal matching—organization in time that is embedded within a spatial context. Each firefly *oscillates*^1^ by repeating its flashing, and individuals synchronize their flashes with their neighbors. To eliminate any potential confusion, when dealing with a process such as the latter, we refer to this as *temporal synchronization*.

**Fig. 1:**
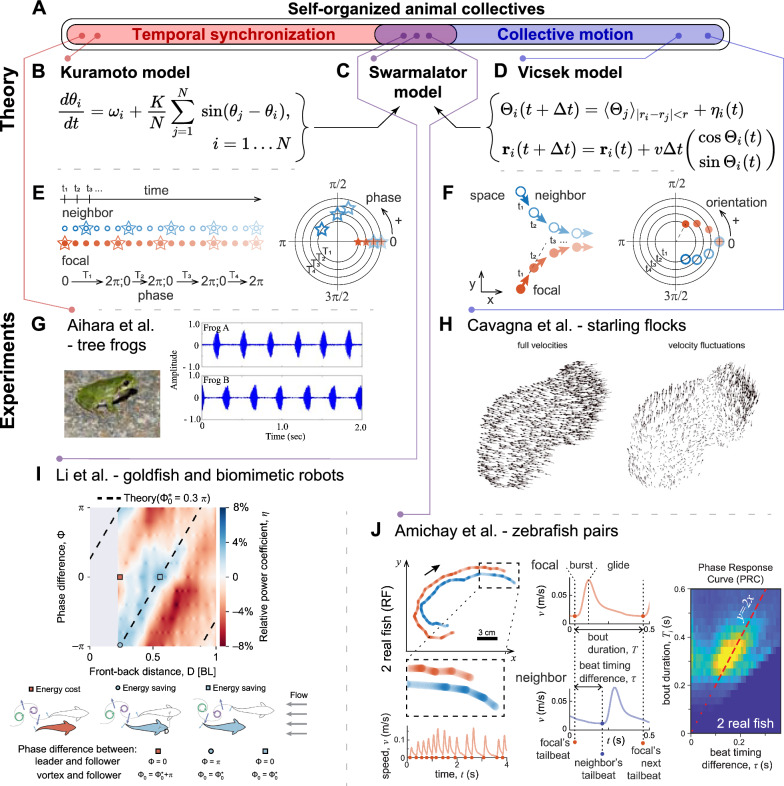
Overview of self-organized animal collectives. **A** Venn diagram of different behavioral ‘classes’ of animal group behavior. **B–D** Theoretical models (Kuramoto [1], Swarmalator [6], and Vicsek [2]), and their principal equations. **E-F** Schematic illustration of the analogy between matching of phases during temporal synchrony and orientation angles during collective motion. **E** Illustration of periodic signals (e.g., flashing, indicated by the star symbol) for two individuals. The focal individual’s periods ($T_{1}$, $T_{2}$, etc.) are used to calculate the relative phase of the neighbor’s signal. On the right, these relative phases are presented on a circle, with separate circles for each period of the focal. **F** Illustration of spatial interaction of two individuals aligning. On the right, the orientations of the individuals are presented as an absolute coordinate on a circle, with the different circles depicting the change in time. **G**-**J** Examples of experimental studies of animal systems belonging to different behavioral classes. **G**: Data from a study on the synchronous behavior of croaking frogs, showing a timeseries of the audio from two frogs, alternating their vocalizations [7]. **H**: Images from a study of collective motion of starling flocks, showing vector fields of the full velocities of all the birds in the flock, and the corresponding velocity fluctuations (the relative velocities with respect to the center of mass velocity). [8]. **I**: Visualizations from a study of spatio-temporal coupling of tailbeats between schooling goldfish (including experiments with biomimetic fish robot pairs)—the heatmap depicting the relative power consumption (energy saving with blue, and extra cost with red) of the robot for different values of the phase difference of the tail undulations vs. the front-back distance of the pair (with a schematic below for intuitive explanation) [9]. **J**: Data from a study of temporal coupling of accelerations between schooling zebrafish [10]. Panels **G**, **H**, **I** and **J** were reused with permission from the corresponding authors as well as the journals. The respective references are provided above

As mentioned, some of the most prominent models in the study of collective behavior include the Vicsek model for collective motion [2] (e.g., flocking, schooling, and herding), and the Kuramoto model for temporal synchronization [1] (e.g., cricket chorusing). In the Vicsek model, individual ‘agents’—abstract representations of individual animals—move by self-propulsion and adjust their heading to match the average direction of nearby neighbors. The Kuramoto model, on the other hand, describes temporal synchronization, or ‘coupling’, among oscillators, such as rhythmic flashing of fireflies.^2^ In both models, individuals align their behaviors with those of others, leading to the emergence of collective properties. While these models might seem quite different, they share a common mathematical foundation: both consider a matching of periodic entities (an angle or a phase) Fig. 1E, F. In the case of flocking, this involves matching headings among individuals across the continuum of 360 degrees in a plane (assuming movement is confined to 2D, which is a plausible simplification that fits the quasi-2D behavior of marching locust nymphs or certain schooling fish; in 3D, there would be two angles to match). Similarly, in temporal coupling, oscillatory processes can be represented on a circle, where repeated behavior over time can be converted into phases wrapped between 0 to 2$\pi$. Thus, here, there is a matching of phases (it is worth noting that the Kuramoto model can also be analyzed in 3D—which would mean a matching of phases on a sphere; or even higher dimensions [11]).

With that in mind, we can view the matching dynamics in these models as analogous (the connection between temporal synchronization and collective motion has been previously established [12]). However, to date, most research has not systematically compared and contrasted these different forms of coordination; besides a few exceptions such as a theoretical framework discussed later [6]). As we will show, at a certain level of description there are commonalities, but at others there are considerable differences. We think that this comparative approach is particularly timely, as increasing attention is being directed towards temporal aspects in animal collectives, highlighted by recent studies [10, 13] and reviews [14]. It is important to emphasize that the aim for unification in this field is not a new objective. For example, the book by Camazine et al. [15], has laid the groundwork for a broad examination of self-organization in living systems. In this work, we focus on two forms of organization, but more in depth.

In what follows, we will provide a synthesis of the seminal empirical and theoretical studies of both fields, along with notable contemporary contributions. We identify mixing dynamics (the switching of neighbors) as a crucial component to understand resulting self-organizing processes, and we will then focus the spotlight on this aspect. We stress the fact that movements need not mean necessarily a high level of mixing; there still may exist (dynamic) structure in many cases. Examination of different types of motion, and how those influence the underlying topologies is of great importance.

## Towards a unified view of spatial and temporal self-organization in animal collectives

We begin by considering how seemingly distinct forms of self-organization actually share commonalities, thereby providing a broader and deeper motivation for this unifying framework. We will emphasize the various terms used across different fields (collective motion/active matter, synchronization/coupled oscillators), which often refer to similar phenomena, highlighting the relevance of this perspective.

When moving in a coordinated manner, animal groups effectively reach a consensus about their direction of travel. This process exemplifies a mechanism of ‘collective decision-making’ [16]. If no individual has a preferred direction, the direction selected by the group is random. If there are a few informed individuals, this can be enough to lead large groups to their preferred direction [17]. Leadership does not necessarily depend on any physical characteristics of the individual, nor does it require individuals to recognize whether others are naive or informed [17]. Directional leadership is akin to temporal entrainment; for example, when entraining an oscillator (such as a firefly), one can ‘lead’ it, to alter its free-running flashes and flash according to the external stimulus provided (though, note that entraining can mean to alter also its’ frequency, and not just phase). How do real-world systems (e.g., croaking frogs) operate in this regard? To the best of our knowledge, mechanisms of ‘temporal leadership’ haven’t been adequately explored.

Collective decision-making processes can also occur in situations where multiple subgroups have different preferences, while the rest of the individuals lack any preferred direction [17, 18]. The proportion of uninformed (or indifferent/naive) individuals can play a counter-intuitive role: increasing the relative number of uninformed individuals can actually increase the likelihood of a collective decision [19]. Similarly, another counter-intuitive result comes from oscillators: it was shown that certain group heterogeneities can, in actual fact, stabilize group synchrony [20]. This suggests that the composition of the group plays a non-trivial role in these dynamics, emphasizing that heterogeneity can be crucial for certain collective behaviors.

There are important differences between the two models. A key distinction is that individuals in the Kuramoto model have frequencies—an element absent in the Vicsek model, which has no analogous attribute. In the Kuramoto model, energy is continuously supplied to each oscillator, resulting in what are known as *self-sustained oscillators.*^3^ Theoretically, this can be disregarded if the oscillators are identical (i.e., have the same natural frequency); in this case, the dynamics can be analyzed from a co-rotating frame of reference.^4^ Experimentally, this aspect remains largely unexplored: for any given real-world system, we still do not know how natural frequencies are distributed within the population (specifically, whether the frequencies can be approximated as equal if the distribution is narrow enough or if there exists a wide range of frequencies). We would like to take a moment here to examine the significance of frequencies in these dynamics more deeply. Multiple real-world examples demonstrate synchrony emerging in groups of individually arrhythmic or even non-periodic individuals, such as neural ensembles [21, 22] or among ants [23–25]. In other words, there may be two separate processes at play: one for the emergence of oscillations and one for synchrony thereafter.

Another vital piece that sets the models apart is the role of noise. In the original Kuramoto model, which is purely deterministic, there is essentially a competition between ‘individuality’ ($\omega$, the first term on the right hand side of the equation in Fig. 1B—which is the natural frequency of each oscillator, that dictates how they would oscillate in isolation) and the ‘conformity’ to the other oscillators (the tendency to match with others)—which depends on the second term, weighted by the parameter *K*. The model can be initialized with some distribution of $\omega$ which determines the heterogeneity of the group. The wider the distribution of $\omega$, the higher the value of *K* must be to counterbalance the disorder created by the heterogeneity and to enable synchronization within the group. It is important to note that this type of disorder is classified as *quenched disorder*, as it is deterministic rather than arising from actual noise (but note that variants of the model that incorporate noise have also been analyzed; see e.g., [26, 27]).

In the Vicsek model, reducing the amount of noise—defined as the directional ‘error’ each individual makes, represented as $\eta$ in Fig. 1D—results in an abrupt change in the collective state. As the noise decreases, the particles transition from random motion to aligned uniform motion in the same (randomly selected) direction. Similarly, when the noise is fixed to a specific value and the density of the particles is increased, a comparable transition from disorder to order occurs [2].

Arguably the most important difference between temporal synchronization and collective motion lies in the aspect of mixing: the dynamical reconfiguration of the underlying interaction network. In collective motion, the movement of individuals themselves changes their relative positions, typically leading to time-varying topologies. By contrast, some synchronous systems (such as certain cricket species) often exhibit more stable topologies with respect to the timescale over which synchronization occurs. The following sections will address the continuum between no dynamical reconfigurations up to systems with very high degrees of reconfiguration over time. The next two sections are organized as follows: each section begins by presenting important and recent contributions with regard to empirical research on both types of coordination, followed by the relevant theoretical work.

## Fixed topologies/neighborhoods

### Experiments: temporal synchrony

Research focusing on empirical studies of synchronous behavior in animal systems includes, for instance, acoustic insects (such as grasshoppers and bush crickets) [28–30] or frogs [7, 31] (see [14] for a recent review on this topic). These studies have revealed that such collectives exhibit relatively local connectivity (i.e., non-global/not all-to-all interactions). Although individuals may be capable of hearing many others—potentially the entire group, depending on its size—they seem to focus on and synchronize with only a select few [14]. However, several open questions remain beyond the specifics of how local these interactions are across different systems. How do these systems manage to achieve the observed global patterns, such as synchrony, waves, and chimera states?^5^ How do individuals respond to their neighbors? Various hypotheses exist; for instance, it could be a competitive scenario in which individuals attempt to be the leading signaler (i.e., the one that calls first, if we are dealing with acoustic signals). Since these are periodic signals, such dynamics can lead to synchrony. Conversely, the interactions may also be coordinated, which could manifest in multiple functional forms. Further studies, particularly those investigating the neurobiological mechanisms involved [32], as well as research on additional species, such as fireflies [13], fiddler crabs [33], or even humans [34, 35] could help shed light on the variety of mechanisms—or, potentially, a few shared common principles—that facilitate the emergence of temporal order in nature.

### Experiments: spatial alignment

In some cases, the underlying network of interaction in collective motion systems can be considered as static. This approach can be appropriate when the behavioral spread occurs much faster than the rewiring of the social network [36–40]. One empirical study examined the rapid spread of alarm responses in schooling fish, characterized by a sudden change in velocity, and thus treated it as a static network. The study demonstrated that the form of contagion was ‘complex’ and the networks of interaction among fish are complex, being weighted, directed and heterogeneous. Furthermore, it revealed that individuals positioned at the front and sides of the group are not only the most influential but also the most susceptible to social influence, as these individuals had relatively few strongly connected neighbors.

### Theory

Static (fixed) networks provide a unique mathematical and theoretical advantage. Given their more stable nature—which, in biological contexts, is always subject to stochasticity and some level of change—these systems can be analyzed theoretically as a continuum (the agents embedded in space, and their level of influence could be dictated by some continuous functional form that is distance dependent) rather than through a discrete-network approach. For certain synchronous systems in which individuals remain mostly immobile while synchronizing, some variants of the paradigmatic Kuramoto model may serve as effective descriptors of the system [41, 42].

Decades of predominantly theoretical research on coupled oscillators have led to prominent progress. Numerical studies have examined finite-size effects (i.e., group sizes that resemble real animal groups) [43]. Certain relaxed variants of the Kuramoto model can be rigorously analyzed using the Ott-Antonsen ansatz, a mathematical tool that reduces the dimensionality of the problem by assuming that the distribution of oscillator phases has a particular form [44]. This enables us, for instance, to gain traction on chimera-forming variants (which may be more relevant to the real-world). However, reality may be more complex—beyond the Kuramoto model of phase oscillators, there exists another class of model where both phase and amplitude are considered (which would mean, for instance, that the magnitude of a firefly’s flashing also conveys information), such as the Stuart-Landau model. Chimera states have been shown to exist also here [45]. Analytical work has allowed researchers to prove the existence of chimeras, which goes beyond numerical evidence [45–47]. As such, there are now attempts to detect chimeras in nature: it was recently argued that certain species of firefly swarms exhibit a chimera state [48].

Another noteworthy class of oscillation models are pulse-coupled and neuron models, which exhibit bursty dynamics as opposed to the smooth *sin* wave dynamics characteristic of the Kuramoto model. Examples include (with decreasing realism): the Hodgkin-Huxley model [49] (which includes detailed ion channel dynamics across the neuron membrane), the Izhikevich model [50] (a reduced description, with only two variables, of the membrane potential and recovery) or the theta model [51] (an even simpler system that replaces membrane potential by a phase variable—the dynamics mapped onto a circle). For detailed descriptions of these models, refer to the aforementioned references, as this is beyond the scope of this paper. Whether or not the assumption of continuous coupling in the Kuramoto model accurately represents reality remains mostly unknown. Although some behaviors appear pulsatile/abrupt, such as a rapid flash of a firefly, this does not necessarily imply that the actual *coupling dynamics* are pulsatile.

## Dynamic topologies/neighborhoods

### Experiments: temporal synchrony

Recent experimental work on synchrony of ‘mobile oscillators’ has focused on the synchronous flash patterns of North American firefly species, which are known to synchronize their flashes while on the move [13]. Much remains to be explored in this area, such as characterizing the movements of the fireflies relative to each other. Is their motion coherent, similar to that of a bird flock, or is it more erratic and random, akin to a swarm of midges [37, 52]? Since firefly trajectories are difficult to fully reveal, it remains unclear whether they form localized clusters, perhaps hovering above an object, or if they disperse through space—coherently or not.

### Experiments: spatial alignment

Returning to collective motion, and assuming predominantly visual interactions, one can computationally reconstruct the visual fields of each individual in a group to reveal who sees whom. Social influence within certain fish groups has been shown to be best described by a visual network, rather than a metric or topological one [53].^6^ Furthermore, it has been found that fish schools alter their network structure by changing their relative spatial positioning, which in turn affects the flow of information within the group [54]. Another important consideration regarding visual networks is that many animals move with variable speed, accelerating and decelerating. These continuous changes also alter their visual scene, occluding neighbors and thus contributing to the temporality of these connections. Recent work has provided further insight into the crucial role of the bi-directional nature of information flow and interaction by utilizing state-of-the-art virtual reality for fish, enabling direct in-situ testing of models [10].

The available ‘social information’ individuals have at any given moment is plentiful. Out of all the neighbors they can sense, they most likely only pay attention to, and are influenced by, a subset of them. Several studies have investigated the selective attention mechanisms involved. For instance, Hinz and de Polavieja demonstrated that fish effectively choose one neighbor at random, subsequently switching to another [55]. Later research revealed that rummy-nose tetra fish swimming dynamics is best explained assuming interactions with two neighbors, and recent work with zebrafish is consistent with this finding [56]). In a slightly different approach, it was shown that fish are most influenced by their faster neighbors [53, 57].

Another noteworthy example of collective motion is found in bird flocks. Seminal papers have employed statistical physics approaches to analyze their collective motion, deriving various correlation functions that provide insights into the hierarchy within the flock [58] or that starling flocks may exhibit ‘scale-free’ properties, implying that regardless of the size of the flock, all birds may indirectly affect and be affected by one another [8].

### Theory: temporal synchrony

We can explore how temporal synchronization might emerge in a group of self-propelled oscillators—oscillators that synchronize their phases while also moving through space, altering their neighborhoods of interaction. How does random motion (i.e., random walks) impact their ability to synchronize? Frasca et al. investigated synchronization among mobile chaotic oscillators (exhibiting irregular and unpredictable oscillations) in 2D and found that movements that enable long-distance changes can actually facilitate the onset of synchronization by increasing the rate at which network configurations switch [59]. Similar findings were observed in 1D motion for Kuramoto oscillators [60]. Continuing this line of research, Fujiwara et al. examined a similar system of chaotic oscillators and discovered that if the timescale of the reconfiguration of the underlying interaction network is much shorter than the timescale of synchronization, the effect of motion can effectively be ignored [61]. Conversely, when the reconfiguration changes occur over much longer timescales, this delays the onset of synchronization [61]. Prignano et al. studied mobile integrate-and-fire oscillators which are ‘pulse-coupled’. They report that at low speeds, local synchronization occurs because each neighborhood remains relatively fixed, leading to global synchronization through multiple interactions between neighborhoods. At high speeds, each oscillator interacts with random neighbors, effectively creating a ‘mean-field’ scenario where global synchronization emerges directly. There exists a mid-range regime where global synchronization cannot be achieved, as both mechanisms fail [62]. It would be of great interest to determine to what extent such a separation of timescales exists in real biological systems.

### Theory: spatial alignment

We now move to the landmark theoretical achievements in collective motion (also known as ‘active matter’ in physics when considering dense groups, where each ‘particle’ is self-propelled and collectively this gives rise to new forms of ‘matter’). The field originated with the publication of the Vicsek model [2] and the continuum version shortly thereafter by Toner and Tu [63], 30 years ago. Further research has led to the discovery of new exotic forms of matter, such as ‘milling’ [64], which has spurred further work generated numerous publications characterizing various collective states and their transitions [65, 66]. These are just a few examples from a very rich body of work; see [67–70] for broad reviews of the field.

The study of collective motion is more ‘mature’ than that of coupled oscillator studies, as theory and experiment have been more closely connected and have informed one another. Therefore, we would like to draw attention to a few select studies that question how effectively these simplified models actually inform us about the biological systems in question—an inquiry that we believe is also central to understanding temporal synchrony.

Multiple approaches have been utilized to derive models consistent with data—with the aim of achieving greater real-world relevance. Bialek et al. analyzed data of European starlings and derived a model based on the maximum entropy principle [71]. The advantage of this approach is that it can be considered assumption free. While it does minimize arbitrary assumptions, there are still decisions to be made—for instance the decision to use normalized velocities which implicitly assumes that their magnitude plays no role. The model they constructed was topological, as opposed to the Vicsek model which is metric. Further empirically grounded work has provided other evidence that also contrasts with Vicsek-type expectations, particularly concerning the lack of ‘behavioral inertia’ (that animals will tend to continue their actions) [37]. Another approach is to ‘zoom out’ from the individual details, and to write down a description of the collective, for example a hydrodynamic theory. Bain and Bartolo treated a crowd of marathon runners as a continuum, and then constrained their model to their observations—the velocity fluctuations in the data appeared longitudinal [72]. Once more, this demonstrated a slight disagreement with the simplified models, in this case with the dynamics of the Toner-Tu model [63]. Our point here is to demonstrate that there is nothing fundamentally wrong with minimal models; however, there is often a need for adjustment or refinement to recover the observed empirical patterns.

### Theory: combined approaches

Finally, due to the mathematical equivalence between the matching of a phase and an angle, theoretical work has considered the Kuramoto model as the framework for collective motion, treating the phase of each oscillator as an angle in space [73, 74]. Similarly, a collective motion model was derived which incorporated a *sin* function for alignment (akin to the *sin* function for phase matching in the Kuramoto model) [75].

A more recent development linking collective motion and synchronization is the study of so-called ‘Swarmalators’—oscillators that not only synchronize but also swarm (i.e., align spatially) [6, 76]; though see also prior work in a similar vain [77–81]. This is essentially a collective motion model with the added complexity that each particle is also an oscillator (without specifying what is actually oscillating, for generality). Particles couple with one another via the Kuramoto model, allowing the two modes of coordination to interact; thus, particles that are synchronized tend to be more spatially attracted to each other. The model generates various collective states and patterns. However, relating such models to real-world systems remains largely unaddressed. Amichay et al. have recently demonstrated that schooling zebrafish temporally couple the timing of their bursty movements in an alternating fashion, which could be considered to be related [10].

In general, the interplay between the matching of angles and phases presents a complex picture, with some results which may be specific to the model employed (i.e., the type of oscillators and the nature and extent of their motion). Can we aspire to achieve a solid theoretical understanding of these self-organizing processes? Are there analytical predictions that extend beyond computational experiments? Yoon et al. have made strides in this direction with the Swarmalator model utilizing the aforementioned Ott-Antonsen ansatz [82]; this could be a promising direction for further research.

## Concluding remarks

Fish schools and firefly swarms, along with many other species, share little in terms of ecological or evolutionary perspectives. However, they exhibit similar collective behavior, stemming from a common mathematical core, making it somewhat surprising that they are seldom considered under the same umbrella. While we acknowledge the theoretical advances made in these fields, we still think that there remains a gap between the studies of collective motion and temporal synchronization, particularly regarding empirical work. Future research could focus on exploring different empirical study systems and comparing how network temporality affects their overall dynamics—such as the differences between less mobile and more mobile firefly swarms. A key objective would be to reveal whether and how motion (i.e., mixing) is detrimental or beneficial for coordination be it temporal or spatial.

As a final note, we argue that the recent advancements in virtual (as well as augmented and mixed) reality technologies offer exciting opportunities for future empirical studies. Researchers could experiment with embedding artificial conspecifics within natural swarms—potentially even in the field—enabling a closer integration of theory and experiment.

## Data Availability

Not applicable.

## Footnotes

1 In other fields such as behavioral ecology, other terms such as ‘repetition rate’ (i.e., tempo/frequency) have been used [4, 5].

2 The Vicsek model is an ‘agent-based’ model, implemented as a computer simulation. The Kuramoto model, by contrast, is a mathematical model that can, in some conditions, have a closed form solution.

3 In the Vicsek model, the agents also have an additional energy supply which they use for self-propulsion.

4 One can disregard frequency by ‘observing’ the dynamics this way; the system reduces to *relative* phases only.

5 Unique spatiotemporal patterns of coexisting synchronization and incoherence that can emerge even among identical oscillators.

6 Individuals focus on a few neighbors somewhat regardless of the distance between them.
