# Publisher Correction: On the integration of collective motion and temporal synchrony in animal collectives

**DOI:** 10.1186/s40462-025-00596-9

**Published:** 2025-09-19

**Authors:** Guy Amichay, Mate Nagy

**Affiliations:** 1 Centre for the Advanced Study of Collective Behaviour, University of Konstanz, Konstanz, Germany; 2 Department of Collective Behaviour, Max-Planck Institute of Animal Behavior, Konstanz, Germany; 3 Department of Biology, University of Konstanz, Konstanz, Germany; 4 Department of Engineering Sciences and Applied Mathematics, Northwestern University, Evanston, IL USA; 5 Northwestern Institute for Complex Systems, Northwestern University, Evanston, IL USA; 6 National Institute for Theory and Mathematics in Biology, Northwestern University, Chicago, IL USA; 7 MTA-ELTE Lendulet Collective Behaviour Research Group, Hungarian Academy of Sciences, Budapest, Hungary; 8 Department of Biological Physics, Eotvos Lorand University, Budapest, Hungary

**Correction to: Movement Ecology (2025) 13:47** 10.1186/s40462-025-00573-2

Following publication of the article, the note on the permission to reproduce figures in the caption of Fig. 1 was captured incorrectly due to a typesetting mistake.

The note originally was captured (incorrectly) as:

The figure we have in the paper contains elements reproduced from other papers. Figure panles **G**, **H**, **I**, **J**, were taken from different published papers.

The correct note should be captured as:

Panels **G**, **H**, **I** and **J** were reused with permission from the corresponding authors as well as the journals. The respective references are provided above.

The original article has been corrected and the publisher apologises to the authors and readers for the inconvenience caused by this error.

